# Scalp acupuncture treatment for children's autism spectrum disorders: A systematic review and meta-analysis

**DOI:** 10.1097/MD.0000000000014880

**Published:** 2019-03-15

**Authors:** Chang Liu, Ting Li, Zhijie Wang, Rui Zhou, Lixing Zhuang

**Affiliations:** Clinical Medical College of Acupuncture, Moxibustion and Rehabilitation, Guangzhou University of Chinese Medicine, Guangzhou, China.

**Keywords:** autism spectrum disorder, meta-analysis, randomized control trials, SAT

## Abstract

**Background:**

Autism spectrum disorder (ASD) is a neurodevelopment disorder without definitive cure. Previous studies have provided evidences for efficacy and safety of scalp acupuncture in children with ASD. However, the efficacy of scalp acupuncture treatment (SAT) in children with ASD has not been evaluated systematically. The objective of this study is to evaluate the efficacy of SAT in children with ASD.

**Methods:**

Information from 6 databases, including MEDLINE, EMBASE, Cochrane database, AMED, China National Knowledge Infrastructure, and Wanfang Data, were retrieved from the inception of each database from 1980 through September 2018. Randomized controlled trials evaluating the efficacy of SAT for patients with ASD were included. The primary outcome measures were the Childhood Autism Rating Scale (CARS) and Autism Behavior Checklist (ABC). The secondary outcome measures were Psychoeducational Profile (Third Edition) (PEP-3) scores. Risk of bias assessment and data synthesis were conducted with Review Manager 5.3 software. Methodological quality was assessed with the Cochrane risk of bias tool.

**Results:**

Fourteen trials with 968 participants were conducted and 11 of the trials were suitable for meta-analysis. Compared with behavioral and educational interventions, SAT significantly decreased the overall CARS scores for children under 3 years old (mean difference (MD) = 3.08, 95% confidence interval (CI) [−3.96, −2.19], *P* < .001) and above 3 years old (MD = 5.29, 95% CI [−8.53, −2.06], *P* < .001), ABC scores (MD = 4.70, 95% CI [−6.94, −2.79], *P* < .001). Furthermore, SAT significantly improved PEP-3 scores in communication (MD = 3.61, 95% CI [2.85, 4.37], *P* < .001), physical ability (MD = 2.00, 95% CI [1.16, 2.84], *P* < .001), and behavior (MD = 2.76, 95% CI [1.80, 2.71], *P* < .001).

**Conclusion:**

SAT may be an effective treatment for children with ASD. Given the heterogeneity and number of participants, randomized controlled trials of high quality and design are required before widespread application of this therapy.

## 1 Introduction

### 1.1 Description of the condition

Autism spectrum disorder (ASD) is a lifelong neurodevelopmental disorder characterized by deficits in social communication and interaction along with restricted and repetitive patterns of behavior, interests, or activities.^[1]^ According to the World Health Organization (WHO), an estimated 1 in 160 children suffer from ASD worldwide, and the prevalence has been rising over the past 50 years.^[2]^ The etiology and mechanisms of ASD are not completely understood.

Although there is no definitive cure for ASD, numerous interventions may improve its symptoms. Treatment modalities for ASD are categorized as behavioral and educational interventions (BEI), psychopharmacologic interventions, or complementary and alternative medicine (CAM).^[3]^ Treatment with risperidone, a common medication for the treatment of maladaptive behaviors in ASD, exhibits adverse side effects such as weight gain, fatigue, drowsiness, and tremors.^[4,5]^ Previous studies have reported that 74% of children with ASD use 1 or more type(s) of CAM treatments because parents of ASD children perceived CAM interventions as safe and natural.^[6]^

### 1.2 Description of the intervention

CAM, which includes acupuncture, is increasingly being applied in children with ASD.^[7]^ Acupuncture has been successfully practiced in China for the last 4000 years and is proven to be tolerable for both ASD children and their parents.^[8]^ A previous review of 27 studies and 1736 patients provides evidence for the efficacy and safety of acupuncture in children with ASD.^[9]^ Among the CAM available, scalp acupuncture treatment (SAT) is widely employed for the treatment of ASD.

SAT is a specialized acupuncture technique involving specific acupoints on a patient's scalp located along different lines or zones. The fundamentals of the SAT were reviewed by Shoukang.^[10]^ In 1993, WHO published the first standard pamphlet on SAT.^[11]^ Research shows that certain hormone levels and cerebral blood flow improve with SAT.^[12]^ Notably, under the influence of neuroanatomy, neurophysiology, and the bioholographic principle of modern medicine, SAT was set up and separated from the traditional acupuncture system in the early 1970s.^[13]^ Controlled trials evaluating the effect of SAT on ASD patients showed significant improvements in language comprehension and self-care ability.^[14]^ The objective of this study was to further evaluate the efficacy of SAT in ASD children.

## 2 Methods

### 2.1 Search strategy

We searched for studies in the MEDLINE, EMBASE, Cochrane, AMED, China National Knowledge Infrastructure, and Wanfang Data databases from 1980 through September 2018. The following search criteria and format were used: (SAT or Scalp acupuncture or Acupuncture or Acupuncture Therapy or Therapy, Acupuncture) and (ASD or autism or Asperger) and (randomized controlled trial or clinical trial or placebo or randomly or trial as topic). Two authors, LC and LT, conducted the search individually. Studies from any language were acceptable. Only published studies were included and conference proceedings were rejected.

### 2.2 Inclusion and exclusion criteria

#### 2.2.1 Research type

Randomized controlled trials (RCTs) comparing an SAT group with at least 10 control intervention were included. There was no limitation on the publication language. Participants with ASD under the age of 18 years were included, regardless of gender or race. Patients were diagnosed according to the Diagnostic and Statistical Manual of Mental Disorders (DSM) or the International Classification of Diseases (ICD). Studies assessed with the Childhood Autism Rating Scale (CARS) or Autism Behavior Checklist (ABC) were accepted, even if diagnostic standards were not mentioned.

#### 2.2.2 Intervention type

The studies chosen used SAT involving the insertion of needles into points on the scalp. Studies using body points as modification and/or pharmacological therapy were excluded. Control interventions included BEI.

#### 2.2.3 Outcome measurement

The primary outcome measures were core features of ASD measured by CARS and ABC. The secondary outcome measures were behavior, physical ability, and communication ability evaluated by Psychoeducational Profile (Third Edition) (PEP-3) scores.

### 2.3 Study selection and data extraction

**Figure 1:**
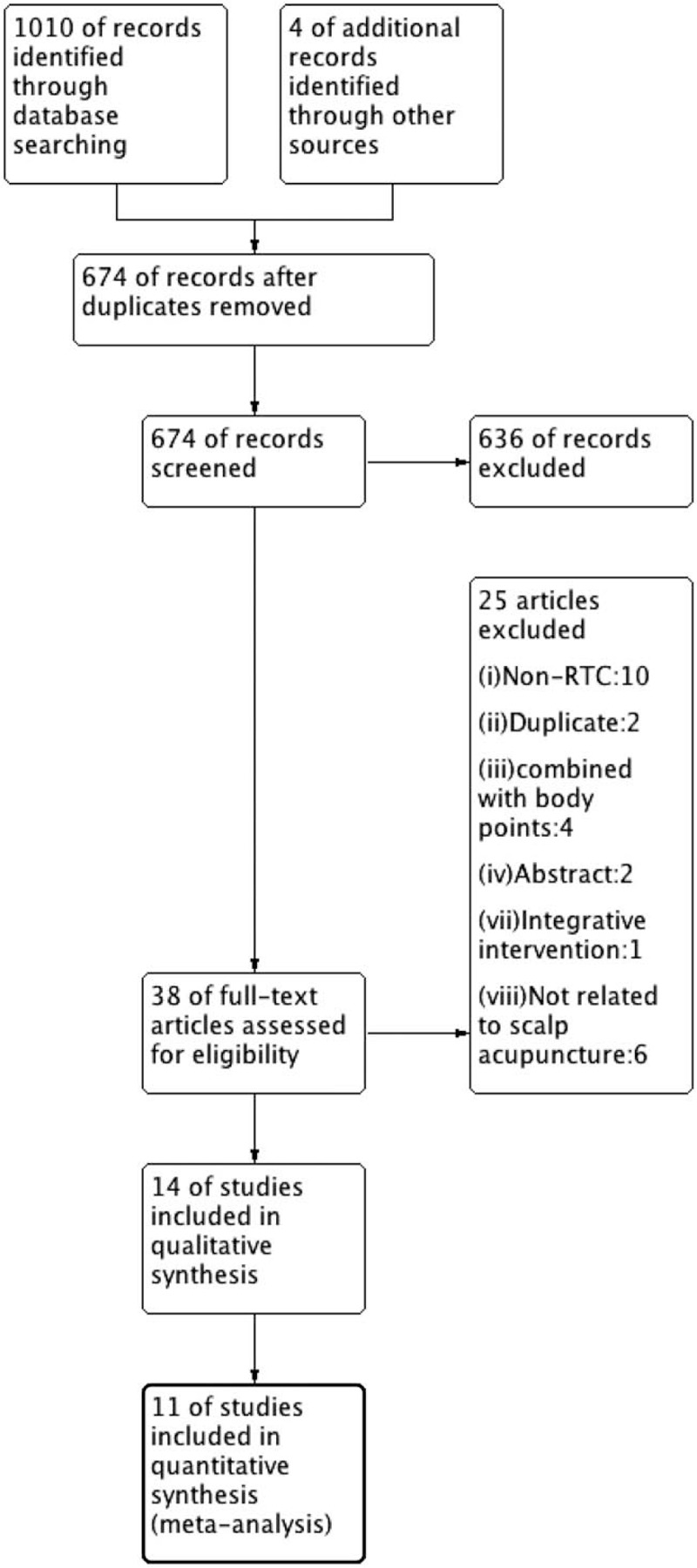
PRISMA flow chart of the literature screening and selection process.

Duplicates of studies were excluded. Two reviewers, LC and LT, independently selected the relevant titles and abstracts of articles. Any disagreements were resolved by consensus with the third author, ZW. If there was any insufficient or missing data in a study, its author was contacted by email. The flow chart of study selection is presented in Fig. 1. A form was filled with the following information: study characteristics (author, published year, sample size, and follow-up), patients’ characteristics (age range, duration of ASD, and diagnostic criteria), details of acupuncture intervention and comparison group (Standards for Reporting Interventions in Clinical Trials of Acupuncture checklist), outcomes (primary and secondary), withdrawals, and adverse events.

### 2.4 Quality assessment

**Figure 2:**
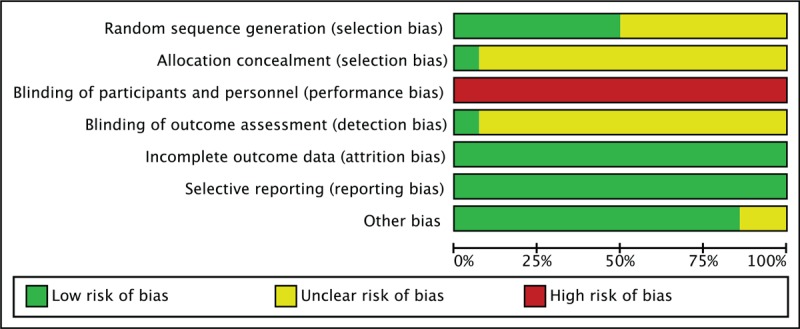
Methodological quality graph.

**Figure 3:**
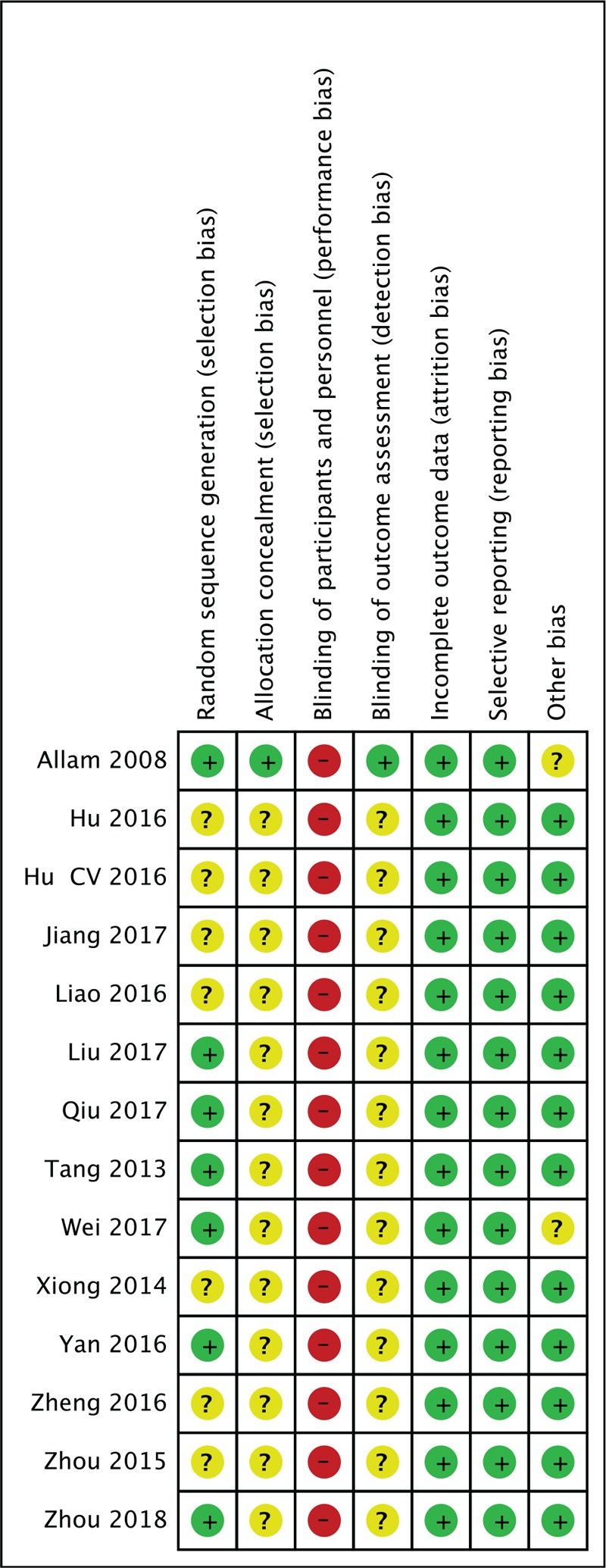
Methodological quality summary.

The Cochrane Handbook version 5.1.0 was used to evaluate the quality of each study.^[15]^ Two authors, ZW and RZ, conducted the evaluation independently. The details of these assessments are described in Figs. 2 and 3.

### 2.5 Statistical analysis

For studies that used the same outcome measure, quantitative synthesis was conducted by meta-analysis with the Review Manager Software version 5.3 (Cochrane, London, UK). Mean difference (MD) and 95% confidence interval (CIs) were used to express continuous data, while risk ratios were used to express dichotomous variables. Heterogeneity of studies was examined by the Higgins *I*^2^ test. A *I*^2^ ≥ 50% was considered substantial heterogeneity and a *I*^2^ ≥ 75% was considered serious heterogeneity. Descriptive analysis was conducted when the number of reported studies was 1. The random-effects model was used when the heterogeneity was significant, whereas a fixed-effects model was used when the heterogeneity was not significant.

### 2.6 Sensitivity analysis

When heterogeneity was significant, studies were excluded in turns, and meta-analyses were repeated. Results were compared, and causes of heterogeneity were discussed.

### 2.7 Assessment of reporting bias

We planned to screen for publication bias using a funnel plot if enough primary studies were available.

## 3 Results

### 3.1 Study description

A total of 1010 studies were identified through the database search, and 4 additional records were added from the reference lists of relevant papers. A total of 340 duplicates were removed, 636 were excluded following review of the titles and abstracts, 38 were included in the full-text assessment, and ultimately, 14 were included in the systematic review, and 11 were included in the meta-analysis (Fig. 1).^[16–29]^

### 3.2 Study characteristics

All trials were single-center RCTs published from 2013 to 2018. Table 1 describes the characteristics of the studies included. Three studies received approval from the institutional review board.^[16,18,19]^ None of the studies registered their protocols before the trial was conducted. Consent forms were obtained in 8 studies.^[16,18,19,21,23,24]^ Two studies conducted follow-ups at 3 and 6 months after treatment.^[22,25]^ CARS was evaluated in 8 trials, ABC was evaluated in 5 trials, and PEP-3 scores were evaluated in 3 trials.^[16–24,26]^ The most frequently used diagnostic criteria was the 4th edition of the DSM-IV-T on the basis of observation. One of the trials diagnosed patients using ICD-10 and Chinese Classification and Diagnostic Criteria of Mental Disorders (third edition).^[16]^ Two of the trials did not report diagnostic methods but did report scores from scales evaluating the core features.^[18,22]^

**Table 1:**
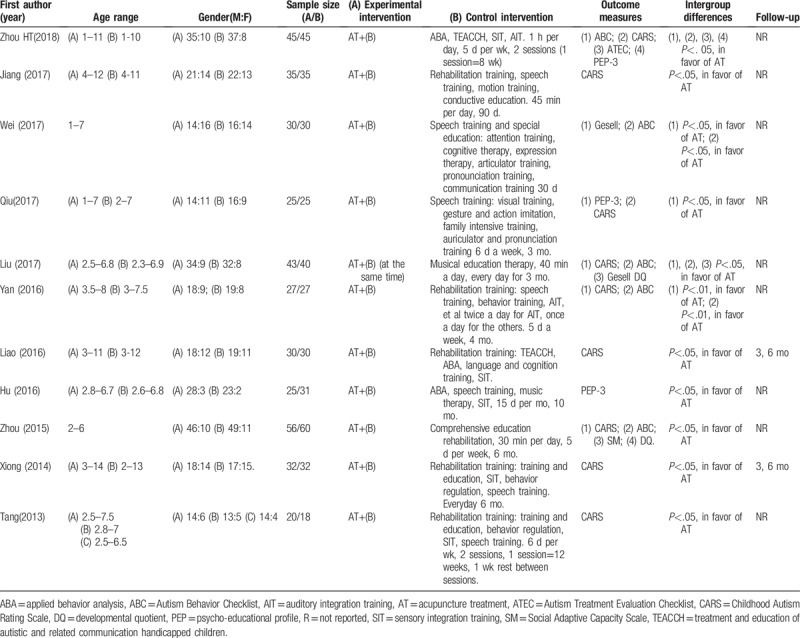
Details of studies include.

### 3.3 Quality assessment

Seven of 11 trials used adequate methods of random sequence generation and were rated as “low-risk.”^[16,18–21,26,28]^ The remaining trials were rated as “unclear” because they did not mention a proper random sequence generation method. ^[17,22–25,27,29]^ Only 1 trial used light-tight envelopes for allocation concealment and blinded the outcome assessors, and was rated as “low-risk” of bias in allocation and assessment.^[28]^ The other studies were rated as “unclear” for this category. It is difficult to blind participants and therapists to acupuncture manipulation procedures. Therefore, all trials were rated as “high-risk” in the performance bias category. All trials reported outcomes completely and properly and were rated as “low-risk” of reporting bias.

### 3.4 Details of experimental interventions

All trials performed manual acupuncture. The most frequently used acupoints were DU24 (5 times), DU17 (4 times), GB19 (4 times), GB13 (4 times), EX-HN1 (4 times), language area (4 times), and ST8 (3 times). Most trials conducted SAT with a depth of insertion ranging from 10 to 25 mm, except for 1 trial that used 1.5 cun (40 mm) on Naosanzhen.^[27]^ Needle retention time ranged from 30 minutes to 8 hours. Nine trials required needle stimulation.^[16,18,20–29]^ Five trials indicated 15 to 20 days of rest between sessions.^[16,18–20,23]^ The course of treatment ranged from 2 to 6 months. Details of interventions are found in Table 2.

**Table 2:**
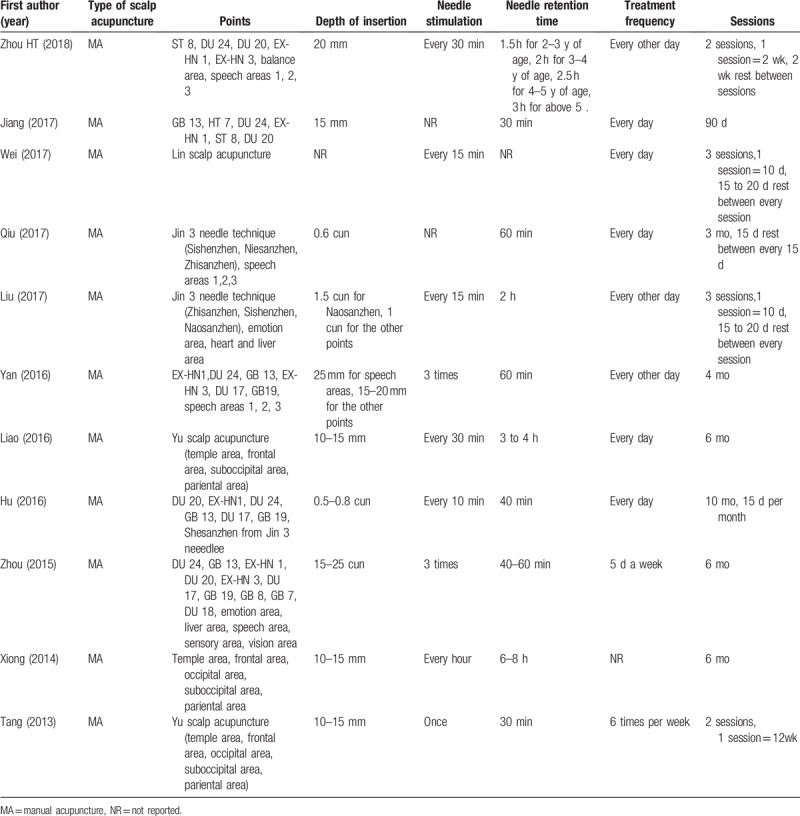
Details of scalp acupuncture.

### 3.5 Efficacy of SAT

#### 3.5.1 Evaluating by CARS, ABC, or PEP-3

**Figure 4:**
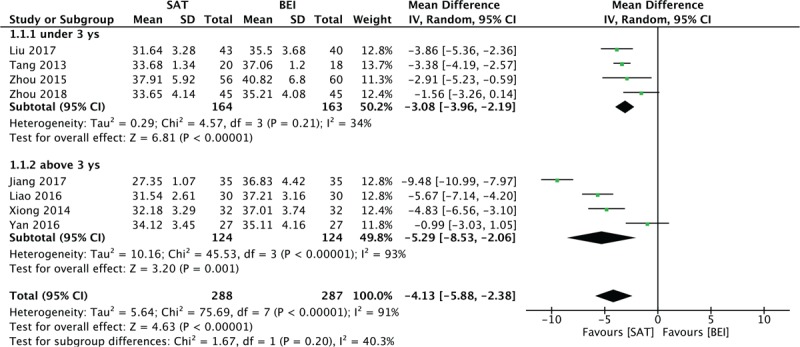
Forest plot for the CARS score. CARS = Childhood Autism Rating Scale.

When grouped together, 8 trials evaluated by CARS exhibited a high heterogeneity of 91% (random model was used). By excluding trials in turns, it was decided that studies with participants of more than 3 years of age should be analyzed in a subgroup. For children under 3 years old, SAT reduced the CARS significantly compared with the control group (MD = 3.08, 95% CI [−3.96, −2.19], I^2^ = 34%, *P* < .001). For children more than 3 years old, the difference was significant (MD = 5.29, 95% CI [−8.53, −2.06], *P* < .001), but with high heterogeneity (I^2^ = 93%; Fig. 4). ABC scores were also reduced compared with the control group (MD = 4.87, 95% CI [−6.94, −2.79], I^2^ = 69%, *P* < .001). PEP-3 scores provided 3 aspects of judgment: communication (MD = 3.61, 95% CI [2.85, 4.37], I^2^ = 0%, *P* < .001), physical ability (MD = 2.0, 95% CI [1.16, 2.84], I^2^ = 0%, *P* < .001), and behavior (MD = 2.76, 95% CI [1.80, 3.71], I^2^ = 0%, *P* < .001). All 3 aspects were significantly increased in the experimental group relative to the control group (Figs. 6–8).

**Figure 5:**
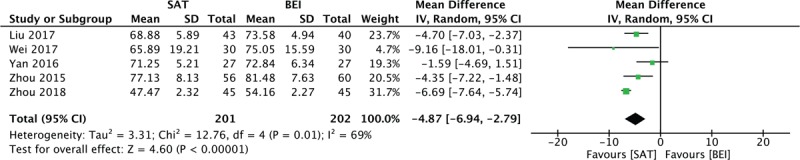
Forest plot for the ABC score. ABC = Autism Behavior Checklist.

**Figure 6:**
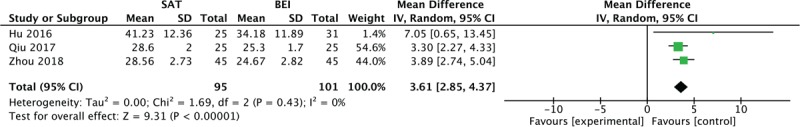
Forest plot for the PEP-3 score in communication. PEP-3 = Psychoeducational Profile (Third Edition).

**Figure 7:**
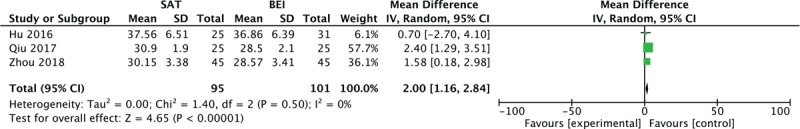
Forest plot for the PEP-3 score in physical ability.

**Figure 8:**
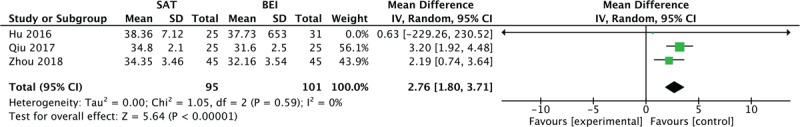
Forest plot for the PEP-3 score in physical behavior.

#### 3.5.2 Evaluating by other scales

One trial suggested that the Autism Treatment Evaluation Checklist Scores in the SAT group were reduced relative to the control group in all aspects (t = 3.72, *P* < .05), including communication (t = 2.71, *P* < .05), social contact (t = 3.06, *P* < .05), sensory and cognitive abilities (t = 3.18, *P* < .05), and health behavior (t = 4.30, *P* < .05).^[16]^ Gesell scores suggested that SAT reduced the developmental quotient (DQ) scores more than the control conditions (t = 2.01, *P* < .05), (t = 2.478, *P* < .01).^[18,29]^ One of the trials only reported portions of Gesell DQ scores, including those in social adjustment (*P* < .05) and social contact (*P* < .05).^[20]^

## 4 Discussion

### 4.1 Main results

This review evaluated 14 RCTs to explore the effect of SAT on ASD. Meta-analysis suggested that compared with BEI, SAT is more effective in decreasing CARS and ABC scores and improving PEP-3 scores in communication, physical ability, and behavior.

### 4.2 Possible explanations

At the time of our literature search, this was the first systematic review and meta-analysis regarding the efficacy of SAT on ASD. None of the trials reported adverse reactions to this therapy. Information of SAT safety requires further research with large sample sizes and long-term follow-ups.

CARS and ABC scores reflect the core symptoms of ASD. Results from a previous study suggest that electroacupuncture increases arginine–vasopressin and oxytocin levels in ASD children, thus improving indifference and passivity.^[30]^ PEP-3 is composed of 10 tests, which includes 3 areas of assessing communication, physical ability, and behavior. Another study showed that acupuncture on GV-20 improves study ability in rats, which may be related to its regulation of postsynaptic density protein-95.^[31]^ The mechanism by which SAT improves these areas requires further research.

### 4.3 Implications

In this review, 13 trials were conducted within 6 months,^[16–22,24–29]^ in which 7 trials were conducted within 6 months.^[16–20,22,26]^ BEI requires more than 6 months to reach efficacy.^[32]^ This implies that the effects of SAT may have an earlier onset than the effects of BEI. High-quality RCTs are needed to definitively reach this conclusion.

It is difficult for children under 3 years old to cooperate for acupuncture on body points in a clinical setting but SAT is easier to conduct, as it does not restrict limb activity. Furthermore, our results suggest that SAT is effective in children under 3 years old. A retrospective study found that the therapeutic effectiveness of SAT decreases with increasing age.^[33]^ One of the trials even suggests that rehabilitation efficacy is improved by retaining the needle on the scalp while training.^[20]^ The effect of age on the efficacy of SAT requires further research.

## 5 Limitations

This review has several limitations. First, most of the trials included exhibited allocation bias and, considering the technique, none of them was able to conduct blinding methods. Second, participants with varying degrees of ASD severity were mixed together, which increases the risk of heterogeneity, and the number of trials was not sufficient to be divided into subgroups.

## 6 Conclusion

In conclusion, SAT may be an effective treatment for children with ASD. Further studies should consider participants’ age and course of the disease. Rigorous and higher-quality RCTs are required in this field.

## Author contributions

LC conceived the study, designed the study protocol, and drafted the manuscript. LC and LT were responsible for study selection. LT and ZW revised the draft. LC and RZ extracted data from articles and evaluated study quality. All authors contributed to the editing of the manuscript and approved the final version accepted for publication.

**Conceptualization:** Chang Liu.

**Data curation:** Chang Liu, Zhijie Wang, Rui Zhou.

**Formal analysis:** Chang Liu.

**Investigation:** Chang Liu.

**Methodology:** Chang Liu, Ting Li.

**Project administration:** Chang Liu.

**Software:** Chang Liu.

**Supervision:** Chang Liu.

**Validation:** Ting Li.

**Visualization:** Ting Li, Rui Zhou.

**Writing – original draft:** Chang Liu, Ting Li.

**Writing – review & editing:** Chang Liu, Ting Li, Zhijie Wang, Lixing Zhuang.
